# Tetra-O-methyl nordihydroguaiaretic acid, an inhibitor of Sp1-mediated survivin transcription, induces apoptosis and acts synergistically with chemo-radiotherapy in glioblastoma cells

**DOI:** 10.1186/1753-6561-7-S2-P8

**Published:** 2013-04-04

**Authors:** Angel M Castro-Gamero, Kleiton S Borges, Daniel A Moreno, Veridiana K Suazo, Mayara M Fujinami, Rosane PG Queiroz, Harley F de Oliveira, Carlos G Carlotti, Carlos A Scrideli, Luiz G Tone

**Affiliations:** 1 Department of Genetics, School of Medicine, Ribeirão Preto, USP, Ribeirão Preto, Brazil; 2 Department of Pediatrics, School of Medicine, Ribeirão Preto, USP, Ribeirão Preto, Brazil; 3 Department of Clinical Medicine, School of Medicine, Ribeirão Preto, USP, Brazil; 4 Department of Anatomy and Surgery, School of Medicine, Ribeirão Preto, USP, Brazil

## Background

Glioblastoma (GBM), one of the most malignant human neoplasms, responds poorly to current treatment modalities, being temozolomide (TMZ) the mostly used drug for treatment. Tetra-O-methyl Nordihydroguaiaretic Acid (M4N) is a global transcriptional repressor of genes dependent of Sp1 transcription factors, such as *Survivin* and *CDK1*. We evaluated expression of *Survivin* and *CDK1* in glioblastoma and analyzed the effects of M4N combined or not with temozolomide and radiation on cell lines and primary cultures of GBM.

## Materials and methods

RT-PCR assays were performed to determinate *survivin-*spliced variants and *CDK1* mRNA expression in glioblastoma tumor samples and cell lines. Cell proliferation was measured by XTT assay, and cell cycle and apoptosis were determined by flow cytometry. Drug combination analyzes using different schedules of administration (simultaneous and sequential) were made based in Chou-Talalay method on GBM cell lines and primary cultures. Gamma radiation for clonogenic survival used the doses of 2, 4, and 6 Gy.

## Results

All *survivin-*spliced variants and *CDK1* gene were expressed in GBM samples (n=16) and cell lines (n=6), except the *survivin-2B* variant that was expressed exclusively in GBM cell lines. M4N decreased cell proliferation separately and synergistically with TMZ, besides enhancement of radiation effects, mainly when associated with TMZ. M4N also induced apoptotic cell death, decreased mitotic index and arrested the cell cycle in G2/M phase. M4N treatment down-regulated *CDK1* gene and *survivin* and s*urvivin-ΔEx3* variants, while the s*urvivin-2B* variant was up-regulated.

## Conclusions

Our results suggest a potential clinical application of M4N in combination with TMZ in GB treatment.

## Financial support

CAPES and FAPESP (process number 2009/50118-2).

